# Optical quality after posterior chamber Phakic implantation of an intraocular Lens with a central hole (V4c implantable Collamer Lens) under different lighting conditions

**DOI:** 10.1186/s12886-020-01340-0

**Published:** 2020-03-04

**Authors:** Tianrui He, Yi Zhu, Jibo Zhou

**Affiliations:** 1 Department of Ophthalmology, Shanghai 9th Peoples Hospital Affiliated to Shanghai Jiaotong, University School of Medicine, No.639, Road Zhizaoju, Shanghai, Huangpu District of Shanghai China; 2 Shanghai Key Laboratory of Orbital Diseases and Ocular Oncology, Shanghai, China

**Keywords:** Implantable collamer lens with a central hole, Optical quality, OQAS, Lighting condition

## Abstract

**Background:**

The current study compared optical quality before and after implantation of a posterior chamber phakic intraocular lens with a central hole [V4c implantable collamer lens (ICL)] under bright and dark lighting conditions by means of the Optical Quality Analysis System™ (OQAS; Visiometrics, Terrassa, Spain).

**Methods:**

This prospective study involved 91 eyes of 46 consecutive high myopia patients (15 males and 31 females, average spherical equivalent − 10.50 ± 0.33D) undergoing implantation of a V4c ICL. The modulation transfer function (MTF) cutoff frequency, Strehl ratio, objective scattering index (OSI), and predicted visual acuities (PVAs; 100, 20, and 9%), under different lighting conditions, were measured before and 1 week, 1 month, and 3 months after surgery.

**Results:**

All optical parameters showed significant improvements, at all time points, under dark condition after surgery. We observed no significant changes in PVA 9% at 1 week (mean value 0.539, *P* = 0.12) or 1 month after surgery (mean value 0.573, *P* = 0.08) under bright condition; but all other postoperative parameters improved significantly. Comparing the two lighting conditions, the OSI decreased more under dark conditions at 1 week (*P* = 0.02), 1 month (*P* = 0.004), and 3 months (*P* = 0.002), but there was no significant difference in any other parameter. In addition, patients were divided into super high myopia (group S, spherical equivalent greater than − 10 D) and high myopia (group H, spherical equivalent from − 6 D to − 10 D), the group S improved significantly more than group H in all parameters, under both bright and dark conditions.

**Conclusions:**

V4c ICL implantation improved optical quality under both bright and dark lighting conditions, and had a better ability to reduce the extent of scattering in the dark. Furthermore, group S achieved greater improvement in visual quality, which should be considered by physicians before surgery.

## Background

A posterior chamber phakic intraocular lens (IOL), the Visian Implantable Collamer Lens (ICL; STAAR Surgical, Nidau, Switzerland), has recently been reported to be an effective, safe, and predictable method for correcting moderate to high myopia [1–7]. However, due to aqueous humor flow and intraocular pressure (IOP), an additional peripheral iridotomy may be needed before or during surgery. A new ICL with a central artificial hole (Visian ICL with Centra FLOW®, V4c; STAAR Surgical, Monrovia, CA, USA) has been developed to resolve this problem, allowing aqueous humor flow through the central hole and thus decreasing the risk of secondary cataract formation and glaucoma [8, 9], as well as avoiding additional hemorrhage and damage to the iris during peripheral iridotomy. Many previous studies have assessed and confirmed the safety, stability, and clinical efficacy of the V4c ICL [10, 11]. Nevertheless, the position of the central hole could affect postoperative optical quality, producing glare, halos, starbursts, and dysphotopsia. There is currently no consensus regarding the optical quality that can be achieved with the V4c ICL. Iijima et al. found that the ICL hole does not induce a significant additional postoperative change in subjective intraocular forward scattering [12]. Kamiya and co-workers, in 2014, also found no significant difference in optical quality between hole ICL and conventional ICL groups [13]. However, Eppig et al. reported that surface reflection from the cylindrical wall of the hole ICL can cause ghosting, as well as additional light spots in peripheral areas [14]. Similarly, Eom et al. reported a hole ICL-induced ring-shaped dysphotopsia, which formed at a retinal field angle of ±40° [15].

However, none of these previous studies mentioned the relationship between optical quality and lighting conditions. Considering the complex relationship between light intensity and the refraction system of the eye, as well as its potential impact on optical quality, it was therefore deemed important in the present study to quantitatively evaluate postoperative visual function under different lighting conditions in the present study.

## Methods

### Design and participants

Consecutive patients requiring ICL implantation to correct high myopia were included in our study. The inclusion criteria were as follows: (1) high myopia with or without astigmatism (manifest SE, − 6.00 D or more); (2) stable refraction within 2 years; (3) a central corneal endothelial cell count > 2000 cells/mm2; (4) an absolute anterior chamber depth (ACD) > 2.8 mm (5) without other ocular or systemic diseases or anomalies (6) without previous or postoperative refractive surgery. Written informed consent was obtained from all participants. The Ethics Committee of Shanghai Ninth Peoples’ Hospital approved the work. All relevant tenets of the Declaration of Helsinki were followed throughout the study.

### Surgical procedure

ICL V4c (Hole ICL™; STAAR Surgical) was used in all eyes included in this study. The ICL power calculation was performed by a modified vertex formula according to the manufacturer. All surgeries were operated following standard procedures [12]. Topical antibiotic agents were used for 3 days before surgery. After pupil dilating and topical anesthesia, ICL was inserted through a 3 mm clear corneal incision. Viscoelastics in the anterior chamber was completely washed out with balanced salt solution, then miotic agent was instilled. After surgery, steroidal (0.1% betamethasone, Rinderon; Shionogi, Osaka, Japan) and antibiotic (0.5% levofloxacin, Cravit; Santen, Osaka, Japan) medications were topically administered four times per day for 2 weeks, with the dose being reduced gradually.

### Optical quality measurement

The modulation transfer function (MTF) cutoff frequency, Strehl ratio, objective scattering index (OSI), and predicted visual acuities (PVAs, 100, 20, and 9%), under scotopic and photopic lighting conditions, were measured using the Optical Quality Analysis System™ (OQAS; Visiometrics, Terrassa, Spain) preoperatively and at 1 week, 1 month, and 3 months after surgery. First, scotopic measurements were performed in a dark room, with the addition of black covers on the instrument, to rule out any influence of light from the computer screen. Second, after turning on the room light source, and a light reflex was induced by shining a penlight (250 lm) into the contralateral eye, photopic measurements were performed. Pupil diameters under both lighting conditions were also measured, with the exception of the OSI (performed for a 4.0 mm pupil); all of the above measurements were performed under the corresponding pupil diameter. Since uncorrected refractive error can directly affect the optical outcome of the system, the manifest refractive errors were fully corrected during these measurements. The spherical error (up to − 8.00 D) was automatically corrected by the double-pass system, and the residual spherical error (over − 8.00 D) and cylindrical error were corrected with an external lens. All measurements were performed three times and the mean value was calculated and recorded. According to measure principle of OQAS, participates with lower OSI, higher MTF cutoff, higher Strehl ratio and higher PVAs tend to have better optical quality, and the determination of the fundamentals and definitions of the parameters have been described previously [13]. The logMAR (logarithm of the minimal angle of resolution) visual acuity and IOP were also measured at each time-point.

### Statistical analysis

All statistical analyses were performed using StatView software (ver. 9.4; SAS, Cary, NC, USA). Generalized estimating equations were used to compare the pre- and postoperative data. The results are expressed as means ± standard error, and a value of *P* < 0.05 was considered statistically significant.

## Results

### Demographic data

A total of 91 eyes (46 patients) were included. All surgeries were uneventful and no intraoperative complication was observed. Table 1 shows the preoperative and postoperative demographic data of the study population. All surgical procedures were uneventful, and no postoperative complication, such as cataract formation, pupillary block, pigment dispersion syndrome, or axis rotation, occurred during the 3-month observation period. No eye was lost during the 3-month follow-up.

**Table 1:** Demographic data of the study population undergoing V4c ICL implantation

Demographic data	
Number of patients (eyes)	46 (91)
Sex (male:female)	15:31
Age (years)	28.46 ± 0.53 (range: 18–44)
Preoperative	
Manifest spherical equivalent (D)	−10.50 ± 0.33 (range: − 21.75 to −5.75)
LogMAR UDVA	1.66 ± 0.04 (range: 1–3)
LogMAR BDVA	0.08 ± 0.01 (range: 0–0.7)
IOP (mmHg)	14.37 ± 0.29 (range: 9–21)
Postoperative (3 months)	
Manifest spherical equivalent (D)	0.03 ± 0.29 (range: −3.23–1)
LogMAR UDVA	0.03 ± 0.04 (range: 0–0.7)
IOP (mmHg)	13.96 ± 0.27 (range: 8–21)

*D* diopters, *logMAR* logarithm of the minimal angle of resolution, *UDVA* uncorrected distance visual acuity, *BDVA* best-corrected distance visual acuity, *IOP* intraocular pressure

### Visual Quality under Different Lighting Conditions.

**Fig. 1:**
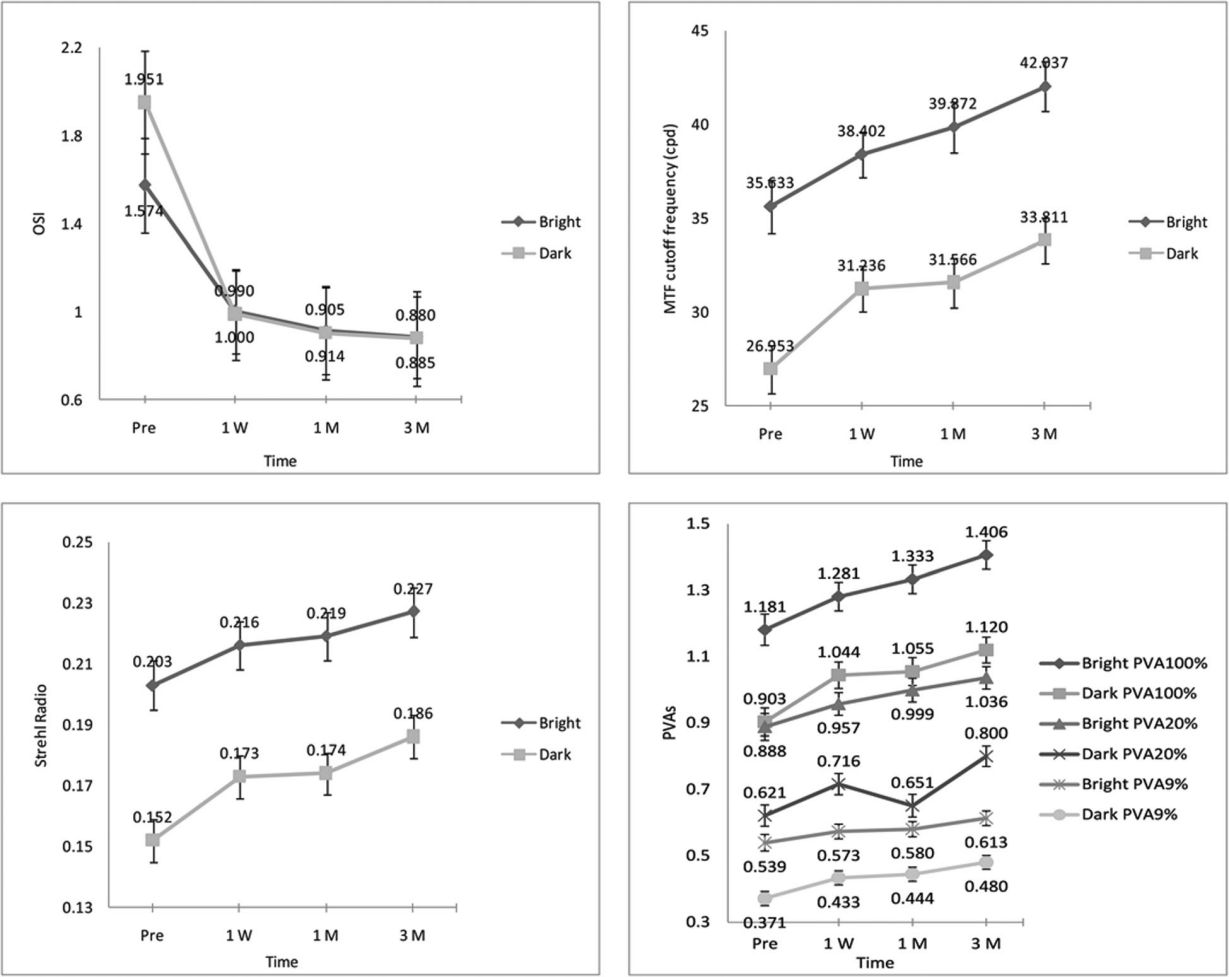
Optical quality preoperatively and 1 week, 1 month, 3 months postoperatively after implantation of posterior chamber phakic implantable collamer lens with a central hole. MTF, modulation transfer function; OSI, objective scattering index; PVA, predicted visual acuity

Figure 1 shows the preoperative and postoperative optical quality. Under both light and dark conditions, all optical quality parameters achieved significant improvement at 3 months after implantation. To further analyze the optical quality data, we measured the changes (postoperative – preoperative, showed as ΔOSI, ΔMTF cutoff, ΔStrehl ratio and ΔPVAs) in each optical quality parameter for each timepoint. Table 2 compared the data obtained under bright and dark conditions. Overall, the V4c ICL performed similarly under both bright and dark conditions, except for the OSI, which showed a greater reduction under dark conditions (*P* = 0.02, 0.004, and 0.002 at 1 week, 1 month, and 3 months, respectively).

**Table 2:** Changes in optical quality parameters in eyes undergoing V4c ICL implantation under different lighting conditions

Time	Parameter	Bright	Dark	*P*-value
1 week	ΔOSI	−0.606 ± 0.131	− 0.920 ± 0.207	0.02
	ΔMTF cutoff frequency (cpd)	2.789 ± 1.133	4.187 ± 1.274	0.22
	ΔStrehl ratio	0.013 ± 0.007	0.019 ± 0.006	0.37
	ΔPVA100%	0.099 ± 0.038	0.131 ± 0.042	0.40
	ΔPVA20%	0.060 ± 0.029	0.086 ± 0.033	0.38
	ΔPVA9%	0.032 ± 0.021	0.051 ± 0.019	0.37
1 month	ΔOSI	−0.686 ± 0.120	−1.034 ± 0.217	0.004
	ΔMTF cutoff frequency (cpd)	4.589 ± 1.109	4.462 ± 1.136	0.91
	ΔStrehl ratio	0.018 ± 0.007	0.019 ± 0.007	0.95
	ΔPVA100%	0.164 ± 0.036	0.143 ± 0.046	0.56
	ΔPVA20%	0.111 ± 0.029	0.113 ± 0.035	0.94
	ΔPVA9%	0.049 ± 0.021	0.052 ± 0.021	0.88
3 months	ΔOSI	−0.689 ± 0.121	−1.056 ± 0.217	0.002
	ΔMTF cutoff frequency (cpd)	6.155 ± 1.207	6.627 ± 1.214	0.70
	ΔStrehl ratio	0.025 ± 0.008	0.031 ± 0.007	0.44
	ΔPVA100%	0.212 ± 0.039	0.209 ± 0.041	0.94
	ΔPVA20%	0.136 ± 0.033	0.162 ± 0.032	0.44
	ΔPVA9%	0.078 ± 0.023	0.094 ± 0.023	0.48

*ICL* implantable collamer lens, *MTF* modulation transfer function, *OSI* objective scattering index, *PVA* predicted visual acuity

### Visual Quality by Degree of Myopia

To determine how the V4c ICL affected the degree of myopia, we further divided the patients into super high myopia (group S, SE greater than − 10 D) and high myopia (group H, SE from − 6 D to − 10 D) groups, and measured changes in optical quality parameters in each group under both bright and dark conditions. There was no significant difference in population, sex, or age between the two groups. Except for the MTF cutoff frequency, Strehl ratio and PVAs at 3 months postoperatively under bright conditions, changes in all parameters in group S were significantly greater than those in group H at all time points, both under bright and dark conditions (Table 3 and Table 4).

**Table 3:** Comparison of changes in optical quality between Group H and S under bright conditions

Parameter	Time	Group H	Group S	P-value
ΔOSI	1 week	− 0.063 ± 0.098	−1.127 ± 0.136	0.005
	1 month	−0.108 ± 0.088	−1.267 ± 0.151	0.003
	3 months	−0.158 ± 0.098	−1.227 ± 0.127	0.004
ΔMTF cutoff frequency	1 week	0.018 ± 1.362	6.042 ± 2.334	0.010
	1 month	1.458 ± 1.450	8.311 ± 2.508	0.006
	3 months	4.465 ± 1.472	8.808 ± 2.393	0.070
ΔStrehl ratio	1 week	0.000 ± 0.009	0.029 ± 0.013	0.028
	1 month	0.001 ± 0.010	0.038 ± 0.015	0.012
	3 months	0.014 ± 0.011	0.038 ± 0.015	0.098
ΔOV100%	1 week	0.011 ± 0.050	0.203 ± 0.080	0.017
	1 month	0.063 ± 0.049	0.281 ± 0.084	0.009
	3 months	0.162 ± 0.049	0.288 ± 0.080	0.117
ΔOV20%	1 week	−0.002 ± 0.045	0.143 ± 0.066	0.029
	1 month	0.038 ± 0.047	0.194 ± 0.071	0.028
	3 months	0.094 ± 0.048	0.199 ± 0.066	0.115
ΔOV9%	1 week	−0.012 ± 0.031	0.087 ± 0.042	0.018
	1 month	−0.006 ± 0.032	0.113 ± 0.046	0.011
	3 months	0.050 ± 0.034	0.111 ± 0.044	0.165

*ICL* implantable collamer lens, *OSI* objective scattering index, *MTF* modulation transfer function, *OV* optical quality analysis system value

**Table 4:** Comparison of optical quality changes between Group H and S under dark conditions

Parameter	Time	Group H	Group S	P-value
ΔOSI	1 week	−0.325 ± 0.121	− 1.606 ± 0.198	0.001
	1 month	−0.379 ± 0.116	−1.746 ± 0.212	0.001
	3 months	−0.429 ± 0.125	− 1.727 ± 0.217	0.002
ΔMTF cutoff frequency	1 week	1.623 ± 1.587	7.443 ± 2.308	0.012
	1 month	1.347 ± 1.593	8.399 ± 2.476	0.004
	3 months	2.994 ± 1.533	10.503 ± 2.274	0.001
ΔStrehl ratio	1 week	0.006 ± 0.008	0.036 ± 0.012	0.011
	1 month	0.006 ± 0.009	0.038 ± 0.013	0.011
	3 months	0.016 ± 0.010	0.048 ± 0.013	0.016
ΔOV100%	1 week	0.058 ± 0.053	0.237 ± 0.077	0.020
	1 month	0.042 ± 0.053	0.275 ± 0.082	0.005
	3 months	0.099 ± 0.051	0.335 ± 0.076	0.002
ΔOV20%	1 week	0.025 ± 0.043	0.169 ± 0.061	0.018
	1 month	0.052 ± 0.042	0.203 ± 0.063	0.016
	3 months	0.089 ± 0.043	0.254 ± 0.060	0.006
ΔOV9%	1 week	0.015 ± 0.026	0.106 ± 0.037	0.013
	1 month	0.019 ± 0.027	0.110 ± 0.038	0.017
	3 months	0.058 ± 0.034	0.137 ± 0.043	0.048

*ICL* implantable collamer lens, *OSI* objective scattering index, *MTF* modulation transfer function, *OV* optical quality analysis system value

## Discussion

In the present study, we assessed the outcome afforded by the V4c ICL according to lighting conditions, and found that it improved optical quality under both bright and dark conditions, while achieving greater improvement in the dark in the S group. In a recent study, Miao et al. measured optical quality at 1 and 3 months after V4c ICL implantation, using the same instrument as in our study, and found no significant difference between the two time points [16]. However, they did not measure the optical parameters preoperatively, nor did they consider the effect of lighting. To the best of our knowledge, this is the first study to objectively assess optical quality in detail under different light conditions after this novel surgical procedure.

During the entire 3-month follow-up, optical quality showed significant improvement under both bright and dark conditions. There could be three explanations for these results. First, the ICL afforded better retinal magnification than spectacles [17]. Kamiya et al. previously reported 1.00 and 0.88-fold improvements in retinal magnification after phakic IOL implantation and use of spectacles, respectively, for correction of high myopia; furthermore, a shrinking image could reduce visual quality [18]. Second, ICL implantation induces significantly higher contrast sensitivity and less spherical aberration [19–21], which could result in better optical quality. Third, single-vision spectacle lenses used to correct myopia could increase hyperopic defocus in the peripheral retina, which may also affect visual quality [22–24].

In the present study, the V4c ICL yielded similar outcomes under both bright and dark conditions, with the exception that the OSI was reduced more under dark than bright conditions; thus, the V4c ICL had stable and excellent performance under different lighting conditions, albeit with a better ability to reduce the extent of scattering in the dark. Due to a lack of relevant previous literature, we can only speculate regarding this increased performance. One possibility is that there is an impact of ring-shaped dysphotopsia, as reported by Eppig and co-workers [14] and Eom and co-workers [15] in 2015 and 2017, respectively, according to a special visual sequela induced by V4c implantation. Both studies suggested that dysphotopsia may be influenced by illumination intensity, and Eom and co-workers reported a subjective feeling in patients that dysphotopsia was more obvious under bright conditions, which may have resulted in less improvement [15]. Furthermore, V4c implantation partially resolves the peripheral hyperopic defocus problem, whereby rod cells receive more signal stimulus, and this could also lead to greater improvement in optical quality. It could also be a sign of deeper problems and should be investigated accordingly.

As expected, we found that patients with super-high myopia experienced greater improvement in visual quality than the high myopia patients, which could be explained by the lower retinal magnification and more serious hyperopic defocus associated with thicker spectacles [22]. Although the differences in MTF frequency, Strehl ratio, and PVAs between the two groups under bright conditions were not significant by 3 months postoperatively, there was a trend toward a better outcome in group S than in group H. The precise reasons of for convergence between the two groups at 3 months need to be studied further. In addition, because many previous studies have shown that ICL yields better outcomes in cases with large refractive errors [25], we propose that ICL implantation should be the first choice for super-high myopia patients.

A limitation in this study was that we did not collect long-term follow-up data. Secondly, because the OQAS only measure monocular optical quality at one time, further binocular visual function evaluations are needed. Thirdly, although the OQAS objectively evaluates optical quality, it cannot measure retina function, yet high myopia patients often show retinal changes. Lastly, we didn’t collect subjective feeling of visual quality during our follow-up, so additional questionnaire measures may be necessary in further researches. Overall, it remains unclear how these differences affect patients’ experience and daily activities.

## Conclusions

In conclusion, the present study showed that optical quality parameters were improved after V4c ICL implantation, and the V4c ICL had a better ability to reduce scattering under dark conditions; this suggests that the optical performance of hole ICL requires further improvement. However, we believe that V4c ICL implantation should play a large role in correction of super-high myopia, and surgeons should be more cautious before performing surgery to correct low-to-moderate myopia, in consideration of cost-effectiveness.

## Data Availability

The datasets for the analysis of the current study are readily available from the corresponding author on reasonable request.

## Abbreviations

BDVA: Best-corrected distance visual acuity
D: Diopters
ICL: Implantable collamer lens
IOL: Intraocular lens
IOP: Intraocular pressure
logMAR: Logarithm of the minimal angle of resolution
MTF: Modulation transfer function
OQAS: Optical Quality Analysis System
OSI: Objective scattering index
PVA: Predicted visual acuity
SE: Spherical equivalent
UDVA: Uncorrected distance visual acuity
